# Case Report: Refractory macrophage activation syndrome requiring high-dose anakinra, emapalumab, and etoposide therapy in early-onset systemic juvenile idiopathic arthritis associated with adenoviremia

**DOI:** 10.3389/fped.2023.1336554

**Published:** 2024-01-22

**Authors:** Elizabeth D. Slaney, Renee Modica, Leandra Woolnough, Dina Kafisheh, Denise Heather Bell-Brunson, Melissa Elder

**Affiliations:** ^1^ College of Medicine, University of Florida, Gainesville, FL, United States; ^2^ Division of Pediatric Allergy, Immunology, and Rheumatology, Department of Pediatrics, University of Florida, Gainesville, FL, United States

**Keywords:** systemic arthritis, macrophage activation syndrome, emapalumab, anakinra, etoposide

## Abstract

Macrophage activation syndrome (MAS) is a life-threatening condition characterized by the excessive stimulation of macrophages and T lymphocytes, provoked by infections, malignancy, and autoimmune or autoinflammatory conditions such as systemic juvenile idiopathic arthritis (sJIA). Clinical signs of sJIA may include high-spiking, quotidian fevers, lymphadenopathy, hepatosplenomegaly, and a salmon-colored migratory, evanescent rash. By contrast, MAS is characterized by unremitting fevers and diffuse, fixed, maculopapular rashes. In addition to hepatosplenomegaly and lymphadenopathy, patients with MAS may also have clinical signs of coagulopathy, as well as cardiac, lung, renal, and central nervous system dysfunction. The empiric treatment for MAS is initially high-dose IV corticosteroids, but usually requires addition of immunomodulators such as tacrolimus or a biologic such as Anakinra to control. The addition of immunotherapies for MAS has improved patient outcomes. We present a 2-year-old male patient with a history of early-onset sJIA, who presented with MAS refractory to corticosteroids and anakinra triggered by adenoviremia that required addition of emapalumab to control. We believe this is the first reported case of a combination of immunosuppressive therapy of emapalumab, etoposide, anakinra, tacrolimus, and corticosteroids used in the successful treatment of infection-induced MAS in early-onset sJIA. Given the lack of treatment guidelines and approved therapies for MAS, alternative strategies should be considered for patients with an intractable course.

## 1 Introduction

Hemophagocytic Lymphohistiocytosis (HLH) is a “cytokine storm syndrome” characterized by the overproduction of proinflammatory cytokines, mainly interleukin 1β (IL-1β), interleukin 6 (IL-6), interleukin 18 (IL-18), interleukin 33 (IL-33), tumor necrosis factor-α (TNFα), and interferon-γ (IFNγ) by activated immune cells (T lymphocytes and macrophages), which leads to life-threatening systemic inflammation. HLH can be primary (genetic or familial) or secondary (triggered by infection, malignancy, or rheumatologic disease). When secondary HLH (sHLH) is associated with an autoimmune/inflammatory disease, it is known as either macrophage activation syndrome (MAS) or “MAS-HLH,” and is estimated to complicate approximately 10%–13% of pediatric patients with systemic juvenile idiopathic arthritis (sJIA) (1, 2). The incidence of subclinical MAS may be as high as 30%. Several known genetic mutations predispose patients to primary HLH (pHLH), including PRF1, UNC13D, STXBP2, STX11, Rab27a, SH2D1A, and XIAP (3–8).

We report the first case of a child with early-onset sJIA with corticosteroid and anakinra refractory MAS triggered by adenoviremia, who required further dose escalation of anakinra (up to 46 mg/kg/day) as well as the addition of emapalumab (up to 10 mg/kg biweekly) and a course of etoposide for the resolution of his symptoms. We believe this is the first case report of this aggressive combination of immunosuppressive therapy and the highest reported dose of non-continuous intravenous anakinra used in the treatment of MAS in the setting of sJIA.

## 2 Case description

The patient is a 2-year-old Caucasian male, ex-33-week premature infant with fetal alcohol syndrome who was diagnosed with early onset sJIA at 13 months of age after he presented with persistent quotidian fevers, evanescent rash, lymphadenopathy, and arthritis. Initial treatment included anakinra 100 mg subcutaneously daily. He had five prior hospitalizations for bouts of MAS, requiring treatment with high-dose intravenous (IV) corticosteroids and IV anakinra. His most recent admission, reported here, was prompted by persistent fevers, rash, laboratory values concerning for MAS flare, and chest x-ray suggestive of multifocal pneumonia. His home regimen prior to admission was 8 mg/kg/day of subcutaneous (SC) anakinra and 0.17 mg/kg/day of tacrolimus (with trough level of 7–9). It was initially unclear whether this episode was a continuation of a previous weeks-long MAS flare or constituted a new flare until results of infectious evaluation revealed extreme adenoviremia.

**Figure 1:**
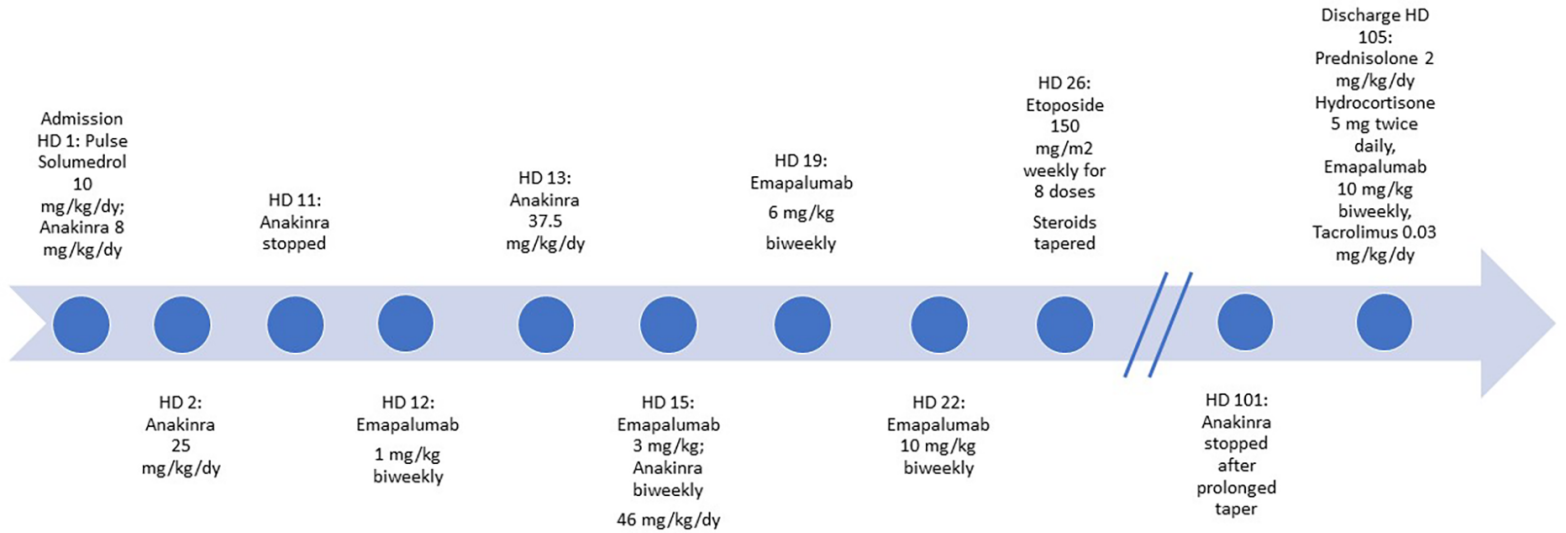
Hospital treatment timeline.

During his admission, which lasted for a total of 105 days, he was initially given pulse IV methylprednisolone (10 mg/kg/day) and was continued on high-dose steroids with inability to taper for weeks. On hospital day (HD) 2, anakinra was increased (25 mg/kg/day IV in divided doses) in the setting of worsening MAS labs. His oral tacrolimus was switched to IV cyclosporine from HD 10–38; however, due to persistently subtherapeutic levels, he was later switched back to oral tacrolimus after developing hypertension. Anakinra 25 mg/kg/day was discontinued on HD 11 to initiate emergent use of low-dose emapalumab 1 mg/kg twice weekly, given worsening MAS labs and fever despite high dose IV anakinra. However, unanticipated issues with IV access delayed his emapalumab infusion and he began to experience spiking fevers as a result. Biweekly emapalumab was added on HD 12 (1 mg/kg) with subsequent weekly dose escalation (3 mg/kg HD 13, 6 mg/kg HD 15, 10 mg/kg HD 19, and 10 mg/kg HD 22 and thereafter). On HD 13, his hyperferritinemia worsened to 67,000 ng/ml, AST/ALT > 1,000 IU/L, LDH >10,000 IU/L, and persistently down-trending platelets, hemoglobin, and leukocyte count. This laboratory trend in conjunction with persistent fevers and worsening respiratory status necessitated increased oxygen supplementation and prompted a transfer to the pediatric intensive care unit (PICU) from HD 13 to 15. Very high dose anakinra was resumed in the PICU (37.5 mg/kg/day IV in divided doses on HD 13). His respiratory deterioration improved after receiving furosemide for interstitial pulmonary edema. He was switched to IV dexamethasone 1.5 mg/kg/day on HD 15. However, owing to worsening MAS labs, his IV anakinra dose was increased further (46 mg/kg/day IV in divided doses from HD 13 to 58). Etoposide (150 mg/m^2^ weekly for 8 doses) was added on HD 26 per pHLH protocol as recommended by pediatric hematology/oncology based on his severe refractory course despite aggressive treatment for sHLH (9). Of note, he did not have a pathogenic mutation in genes responsible for pHLH. He eventually stabilized after nearly 3 months of this combination therapy plus cidofovir for his adenoviremia, facilitating slow taper of high-dose IV steroids as well as anakinra from 46 mg/kg/day to 2 mg/kg/day over the course of 37 days (HD 59–96). Anakinra was eventually discontinued on HD 101. At discharge (HD 105), he was prescribed prednisolone 2 mg/kg/day, hydrocortisone 5 mg twice daily, emapalumab 10 mg/kg twice weekly, tacrolimus 0.2 mg twice daily (0.03 mg/kg/day) (Figure 1), as well as maintenance cidofovir.

As noted above, evaluation for pHLH was negative by genotyping of blood and bone marrow. Invitae^©^ primary immunodeficiency genetic panel, quantitative immunoglobulins, and lymphocyte subsets were normal and did not indicate an underlying immunodeficiency. Bone marrow biopsies (at initial diagnosis and with current MAS flare) did not demonstrate malignancy and comprehensive infectious evaluation was entirely negative other than adenoviremia (>10,000,000 copies/ml). He was treated with weekly IV cidofovir, which was continued after discharge home, and five doses of IV immunoglobulin G (IVIG) to provide passive immunity. Chest computed tomography (CT) confirmed multifocal pneumonia and excluded interstitial lung disease. He was treated with voriconazole for presumed Aspergillus because of abnormal galactomannan from bronchoalveolar lavage (BAL), which was converted to posaconazole at discharge. However, there remains a debate whether he in fact actually had fungal pneumonia given that only one BAL galactomoannan was abnormal with a negative result on repeat BAL. Repeat BAL universal polymerase chain reaction (PCR) revealed *Pneumocystis jiroveci* (PJP) despite prophylaxis with nebulized pentamidine, which was treated with atovaquone (Table 1).

**Table 1:** Clinical work-up for HLH.

Category	Labs	Pathology	Imaging
Malignancy	HLH gene mutations: *negative* Invitae© primary immunodeficiency panel: *negative* Peripheral blood flow cytometry: *negative for malignant cells*	Bone marrow aspirate: *negative for leukemia × 2 (at initial diagnosis and with current flare)* Inguinal lymph node biopsy: *reactive cells without malignancy*	CT chest scan: groundglass airspace opacities, reactive mediastinal adenopathy
Infections	Adenovirus PCR: positive (>10,000,000 copies) (1,3)-beta-D-glucan: positive HHV6 PCR: detected HIV: *negative* SARS-COV2: *negative (PCR and IgG M-spike)* Quantiferon: *negative* Influenza A/B, EBV, CMV, Parvovirus, Norovirus, Hepatitis A/B/C, Rhinovirus: *negative*	Bronchoalveolar lavage: abnormal galactomannan and universal PCR Pneumocystis jiroveci Urinalysis: *normal* Stool studies: *no pathogens detected*	CT chest scan: multifocal pneumonia
Autoimmune and autoinflammatory	IL-13, IL-10, IL2R, IL-17, IL1beta, IL-6, TNF-α, CXCL9, IL-18: elevated IL-2, IFNγ, IL-4, IL-5, IL-8: *within normal limits*		Echocardiogram: *negative for coronary artery dilation or pericardial effusion*

Now 24 months post hospital discharge, he has not had recurrence of MAS off steroids, anakinra, and emapalumab, remaining only on low dose tacrolimus and methotrexate for treatment of his sJIA. He receives monthly inhaled pentamidine for *Pneumocystis jirovecii* pneumonia (PJP) prophylaxis. He continues to be followed by Pediatric Rheumatology/Immunology for further management.

## 3 Discussion

MAS is a potentially life-threatening, hyperinflammtory syndrome that may complicate sJIA and lead to other autoimmune disorders resulting in hemophagocytosis primarily involving the bone marrow, liver, and spleen; however, hemophagocytosis can be seen elsewhere such as in the cerebrospinal fluid (CSF) (1). The overlapping clinical features of active sJIA and MAS may delay the diagnosis, and therefore application of the 2016 Classification Criteria for MAS complicating sJIA may be helpful in distinguishing between these conditions (1, 10). The clinical features of MAS include unremitting high fevers, lymphadenopathy, hepatosplenomegaly, maculopapular rash, coagulopathy, renal dysfunction, altered mental status, and may involve other organs as well. Laboratory features include evolving cytopenias, elevated C-reactive protein, precipitously declining erythrocyte sedimentation rate, hypofibrinogenemia, transaminitis, elevated lactate dehydrogenase (LDH), hypertriglyceridemia, hyperferritinemia, and hemophagocytosis on bone marrow biopsy (2). There are no standardized treatment protocols for MAS. Typically, it is managed empirically with high-dose IV corticosteroids, a calcineurin inhibitor, and a biologic. The addition of a variety of novel immunotherapies designed to inhibit specific cytokines involved in the activation of the immune system has improved outcomes for patients with MAS. Anakinra, a recombinant IL-1 receptor antagonist, is efficacious in the treatment of MAS at doses of 1–2 mg/kg/day in children (11–13). More recently, higher doses of anakira have been used in the 4–10 mg/kg/day range.

Although no formal diagnostic criteria or management guidelines exist for treatment of refractory MAS, multiple therapeutic approaches are well described in the literature. Anakinra is not always effective at standard dosing for unremitting MAS in sJIA patients and may require doses >10 mg/kg/day or 2 mg/kg/hour in children with sHLH/MAS. A dose of 2,400 mg/day as a continuous IV infusion was required in a cohort of critically ill adults with MAS (14, 15). Emapalumab, an IFNγ-blocking monoclonal antibody, is effective in the treatment of pHLH, although its use in sHLH/MAS has yet to be approved and is under investigation (16–18). INFγ and IFNγ-related chemokines (CXCL9, CXCL10, CXLC11) levels are notably higher in patients with active sHLH/MAS and are tightly associated with laboratory markers of disease severity (18–20). These studies suggest that IFNγ is a potent driver of sHLH/MAS. Emapalumab has been shown to rapidly neutralize IFNγ and IFNγ-related cytokine levels and has resulted in MAS remission (18, 20–22). Notably, our patient had elevated CXCL9 and IL-18 levels and responded well to emapalumab.

Etoposide, a topoisomerase-II inhibitor, has been employed in the treatment of unrelenting MAS/sHLH (23). Apheresis has been used to manage and induce remission in refractory MAS in patients with sJIA through the removal of proinflammatory cytokines and reduction in peripheral white blood cells (24, 25). Tofacitinib, a Janus kinase inhibitor, which blocks intracellular signaling thereby diminishing the inflammatory cascade, was successfully used in refractory adult-onset Still's disease complicated by MAS, but is now used sparingly (26). The IL-6 inhibitor, tocilizumab, has been used to safely and effectively treat sJIA with refractory MAS (27, 28).

Our patient did not respond to therapeutic doses of cyclosporine or tacrolimus in combination with high-dose steroids (methylprednisolone or dexamethasone) and escalated doses of anakinra. Therefore, emapalumab and etoposide were added for critical worsening of MAS. Our patient's MAS was driven in large part by adenoviremia; however, he did not dramatically respond to initial cidofovir until steroids were tapered. Although plasmapheresis has been used as rescue therapy in refractory MAS, this option was deferred owing to concerns regarding cardiovascular status. Fortunately, he stabilized on this multifaceted regimen and anakinra was discontinued before discharge. He was weaned off emapalumab after 16 months of maintenance treatment. The patient remains on low dose tacrolimus, but steroids were discontinued 4 months after discharge*.* He was able to discontinue cidofovir after 21 weeks of treatment and his posaconazole prophylaxis for Aspergillus was discontinued after 18 months but he remains on pentamidine prophylaxis. He has not had any flares of MAS since discharge. However, he sustained some infections including norovirus, enteropathogenic *E. coli*, and *Legionella* bacteremia treated with azithromycin. He briefly seroconverted positive with low level adenoviremia that responded to an additional dose of cidofovir without recurrence of MAS.

The cytokine storm seen in recalcitrant MAS can cause life-threatening multiorgan failure, in which IFNγ plays a key pathogenic role. Promising, essential, and innovative cytokine-blocking therapeutic approaches that quell the immune dysfunction seen in these hyperinflammatory states are being investigated. Of these therapies, emapalumab, an IFNγ blocking monoclonal antibody, proved efficacious in this patient over steroids, tacrolimus, and anakinra. Given the extreme cytokine cascade in these conditions, high-dose anakinra in combination with other anticytokine and immunosuppressive therapies (such as etoposide) may have a role in treating gravely ill patients who are refractory to standard dosing and/or other therapeutic interventions. Potentially, this combination approach may assist with an expedited laboratory and clinical recovery in critically ill pediatric patients with recalcitrant MAS.

Our patient had no adverse side effects from this treatment regimen other than possible opportunistic pulmonary infections, which were successfully treated. His severe adenoviremia was the precipitating factor in his MAS flare and cidofovir was necessary adjunct therapy. Given the lack of treatment guidelines and approved therapies for refractory MAS, alternative and aggressive management strategies need to be considered for patients with an intractable course.

## Data Availability

The original contributions presented in the study are included in the article/Supplementary Material, further inquiries can be directed to the corresponding author.
